# Efficacy and safety of epigallocatechin-3-gallate in treatment acute severe dermatitis in patients with cancer receiving radiotherapy: a phase I clinical trial

**DOI:** 10.1038/s41598-023-40881-4

**Published:** 2023-08-24

**Authors:** Jingjing Xie, Li Jia, Peng Xie, Xiaoyan Yin, Wanqi Zhu, Hong Zhao, Xin Wang, Xiangjiao Meng, Ligang Xing, Hanxi Zhao, Xiaolin Li

**Affiliations:** 1 Department of Radiation Oncology, Shandong First Medical University Affiliated Cancer Hospital (Shandong Academy of Medical Sciences), Jinan, 250117 Shandong China; 2 Department of Radiation Oncology, The Fourth People’s Hospital of Jinan, Jinan, 250031 Shandong China

**Keywords:** Radiotherapy, Quality of life

## Abstract

To evaluate the safety and effectiveness of epigallocatechin-3-gallate (EGCG) solution treating the acute severe dermatitis in patients receiving radiotherapy. This phase I research enrolled patients with thoracic cancer receiving radiotherapy at Shandong Cancer Hospital and Institute in Shandong, China. EGCG solution was sprayed to the radiation field when grade III radiation-induced dermatitis (RID) first appearance. EGCG concentration escalated from 660 to 2574 μmol/L using modified-Fibonacci dose-escalation. RID and related symptoms were followed up every day. Between March 2021 and November 2021, 19 patients were enrolled in this phase I research. The median dose of grade III RID first observation was 44 Gy (30.6–52 Gy). As the EGCG treatment was performed continuously, all these grade III RID reactions were significantly decreased to grade I or grade II RID at three days after use of EGCG (*p* < 0.001). Significant relief can be observed in burning sensation (*p* < 0.001), tractive sensation (*p* < 0.001), tenderness (*p* < 0.001), erythema (*p* < 0.001), itching (*p* < 0.001) and pain (*p* < 0.001) after 15 days of EGCG treatment. No radiation therapy delay or interruption for all 19 patients. No adverse events were observed and reported associated with EGCG. The highest dose of this Phase I trial (2574 μmol/L) was recommended for continuous Phase II trial for further evaluation. In this phase I clinical research, use of EGCG solution is safe and can significantly relief grade III RID in patients receiving radiotherapy. Thus, EGCG might be a new choice for acute sever RID.

**Trial Registration:** ClinicalTrials.gov Identifier: NCT02580279 (Full date of first registration: 12/2014).

## Introduction

Radiation therapy (RT) remains an important treatment of thoracic cancer, such as locally advanced non-small cell lung cancer^1^ (NSCLC), limited stage small cell lung cancer^2^ (SCLC), esophageal cancer^3^, breast cancer^4^ and mesothelioma. Most of these cancers require radiation therapy as adjuvant therapy, definitive treatment or as palliative intent. Within last 5 years, the technology of radiotherapy has developed rapidly: including Cyberknife^5^, Image-guided Intensity-modulated Radiotherapy^6^, Unity MR-Linac^7^, TomoThreapy^8^ and even Proton radiotherapy^9^. These precise radiotherapy technologies make radiation dose escalation possible.

The most common adverse event associated with thoracic cancer radiotherapy is radiation-induced dermatitis^10^ (RID), especially in supraclavicular region. RID might manifest as an initial transient erythema that can occur within the first 24 h after RT. With the cumulative radiation dose reaches 18–20 Gy, dry desquamation can develop^10^. While moist desquamation can be seen when RT dose higher than 40 Gy, which is more severe and painful^10^. What’s worse, infection and ulcer formation can stop RT till the area is able to re-epithelialize and heal^11^.

There is no standard effectual remedy to severe RID. Our research group previous carried out a phase 2 randomized clinical trial to evaluate the efficacy of epigallocatechin-3-gallate (EGCG) to prevent grade I to II RID of patients with breast cancer^12^. Results showed that prophylactic use of EGCG solution significantly reduced the incidence and severity of RID^12^. Thus, EGCG has the potential to be a new choice of treatment severe RID for patients receiving radiotherapy.

However, previous studies^12,13^ have mainly focused on patients with no skin ulcers below grade 2. Based on the good results of EGCG prevention of severe RID in our previous experiment, we conducted this phase I dose escalation study for patients with grade 3 RID. The safety and efficacy to treat sever RID of different EGCG concentrations were analyzed.

## Methods

### Epigallocatechin-3-gallate administration

EGCG (purity ≥ 95% by high performance liquid chromatography) was purchased from HEP Biotech Co., Ltd (Ningbo, Zhejiang, China) and freshly dissolved in 0.9% saline solution. The EGCG concentration escalated from 660 μmol/L, 1320 μmol/L, 1980 μmol/L, 2574 μmol/L. EGCG administration was initiated when Grade 3 RID occurred first time during radiation therapy. The solution was sprayed three times a day at 0.05 mL beyond the whole radiation field until 2 weeks after first day use of EGCG. Skin wrinkles in armpits and chest wall, need to be fully stretched and exposed before spraying. Other preventive and treatment agents such as: deodorants, lotions, creams, perfumes, external preparations are not allowed in the radiation field during radiation therapy.

### Study design and participants

Our research team prospectively conducted this research at department of thoracic radiotherapy of Shandong Cancer Hospital and Institute in Shandong Province, China. The inclusion criteria included: (1) patients 18 years or older; (2) patients with histologically confirmed breast cancer, lung cancer or esophageal cancer. (3) Patients received definitive radiation therapy in which the target included supraclavicular area. (4) Eastern Cooperative Oncology Group performance status of 0–1. 5. Adequate hematologic, hepatic, and kidney function profile. (5) Chemotherapy, targeted therapy and endocrine therapy are allowed.

Exclusion criteria included: (1) patients with unhealed wounds in the radiation area. (2) Patient has severe uncontrolled systemic diseases, such as acute circulatory diseases; severe pneumonia, etc. (3) Women of childbearing age need to exclude pregnancy or lactation. (4) The patient is allergic to EGCG-related components.

This study has been approved by the Ethics Committee of our research center (Shandong First Medical University Affiliated Cancer Hospital). Informed consent was obtained from all patients and/or their legal guardian(s). All methods were performed in accordance with the relevant guidelines and regulations.

### Radiotherapy

Patients were simulated by a big-bore computed tomography. Patient imaging data were transferred to the Eclipse treatment planning system (Eclipse 8.6, Varian Medical Systems) for target delineation and radiation planning. The plan was designed using 6-MV beams with IMRT plan for all patients. The prescribe radiation therapy dose were detailed in results Table 1.

**Table 1:** Radiation therapy characteristics of patients.

Characteristics	No	%
Radiotherapy field		
Chest + supraclavicular	4	21.05
Chest + supraclavicular + IM	1	5.26
WB + boost	1	5.26
WB + supraclavicular	1	5.26
supraclavicular	5	26.32
Supraclavicular + MLN + Te	3	15.79
supraclavicular + MLN + Tl	4	21.05
RID evaluated area		
Supraclavicular area	16	84.21
Axilla	3	15.79
Radiotherapy dose		
50 Gy in 25 fractions	8	42.11
44 Gy in 22 fractions	1	5.26
59.4 Gy in 33 fractions	6	31.58
60 Gy in 30 fractions	3	15.79
47.88 Gy in 18 fractions	1	5.26

*S* supraclavicular area, *IM* internal mammary lymph nodes, *WB* whole breast, *MLN* mediastinal lymph node, *Te* primary tumor of esophageal cancer, *Tl* primary tumor of lung cancer.

### Evaluation of RID and RID-related symptom

RID was evaluated and recorded every day by independent 2 observers according to the RID grade of Radiation Therapy Oncology Group (RTOG).

Radiation Therapy Oncology Group score: Grade Description: 0 No change over baseline;1 Follicular, faint or dull erythema/epilation/dry desquamation/decreased sweating; 2 Tender or bright erythema, patchy moist desquamation/moderate edema; 3 Confluent, moist desquamation other than skin folds, pitting edema 4 Ulceration, hemorrhage, necrosis. Grade 0 is no radiation-induced dermatitis. Grade 1 is considered a mild score. Grade 2 is considered a moderate score, and Grade 3–4 is considered a severe score. RID-related symptoms included erythema, burning feeling, itching, pulling, and pain were also evaluated according to Skin Toxicity Assessment Tool every day.

### Safety assessments

Adverse events were evaluated and recorded every three days. All adverse events correlated with EGCG were recorded according to CTCAE, 5.0. These include but are not limited to drug-induced skin rash, nausea and vomiting and other gastrointestinal reactions, fever, palpitation, chest tightness, suffocation and other symptoms. At the same time, we should also distinguish whether the above symptoms are related to EGCG or radiotherapy and chemotherapy.

### Statistical analysis

Statistical analysis was performed using SPSS. The correlation between variables was analyzed using the Spearman rank correlation test. Continuous variables were compared using the t-test. All tests were two-sided, and p values of less than 0.05 were considered statistically significant.

### Ethical approval

IRB approved Identifier from the Ethics Committee of Shandong First Medical University Affiliated Cancer Hospital.

## Results

**Figure 1:**
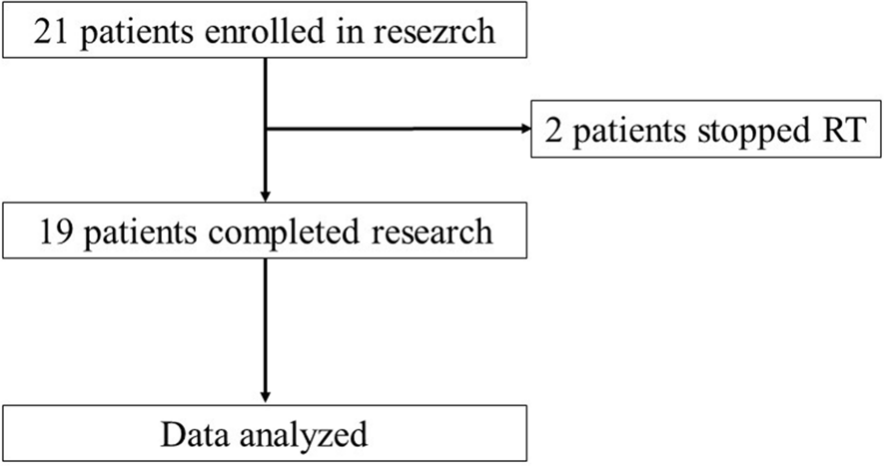
Clinical trial profile.

Between March 2021 and November 2021, 21 patients were enrolled and 2 patients stopped radiation therapy. Data of 19 patients were analyzed in our research (Fig. 1). The clinical characteristics of all eligible patients were summarized in Table 2.

**Table 2:** Clinical characteristics of patients.

Characteristics	No	%
Age, mean (SD)	53 ± 10.849	
Range	29–74	
ECOG		
0	7	36.84
1	12	63.16
2	0	0
Gender		
Female	10	52.63
Male	9	47.37
Tumor type		
Breast cancer	8	42.11
Lung cancer	4	21.05
Esophageal cancer	7	36.84
Pathology type		
Infiltrating ductal carcinoma	8	42.11
Squamous carcinoma	7	36.84
Small cell lung cancer	1	5.26
Non-small cell lung cancer	3	15.79
Stage		
I	0	0
II	4	21.05
III	9	47.37
IV	6	31.58
Treatment		
RT + CT	13	68.42
RT + CT + ET	6	31.58

*RT* radiation therapy, *CT* chemo therapy, *ET* endocrine therapy.

### Effect of EGCG for RID

During the observation of all patients, grade I RID occurred in all patients. The earliest appearance time of grade I RID among our sample was two weeks after radiation therapy (20 Gy/10 fractions), with a median grade I RID dose of 34.58 Gy (range 20–41.4 Gy). The last time grade I RID was observed in an esophageal cancer patient at the sixth week with RT dose of 41.4 Gy/23 fractions. The median dose of grade III RID first observation was 44 Gy (range from 30.6 to 52 Gy). Radiation dose and fractions of patients’ first observations of grade III RID were detailed in Table 3. At the point of first observation grade III RID for each patient, EGCG was prescribed for each patient after carefully check by oncology radiologist.

**Table 3:** Epigallocatechin-3-gallate (EGCG) treatment and RID scoring.

No. of patients	RT dose of grade I RID	RT dose of grade III RID	EGCG dose (μmol/L)	EGCG treatment time (weeks)
1	40 Gy/20f	50 Gy/25f	660	3
2	39.6 Gy/22f	48.6 Gy/27f	660	3
3	41.4 Gy/23f	48.6 Gy/27f	660	3
4	30 Gy/15f	42 Gy/21f	1320	3
5	24 Gy/12f	46 Gy/23f	1320	2
6	24 Gy/12f	42 Gy/21f	1320	3
7	36 Gy/20f	45 Gy/25f	1980	3
8	21.6 Gy/12f	30.6 Gy/17f	1980	4
9	30 Gy/15f	36 Gy/18f	1980	4
10	21.6 Gy/12f	41.4 Gy/23f	1980	4
11	36 Gy/18f	44 Gy/22f	1980	3
12	20 Gy/10f	32 Gy/16f	1980	4
13	30.6 Gy/17f	37.8 Gy/21f	2574	4
14	40 Gy/20f	52 Gy/26f	2574	3
15	30 Gy/15f	38 Gy/19f	2574	3
16	34.58 Gy/16f	39.9 Gy/15f	2574	3
17	36 Gy/18f	50 Gy/25f	2574	3
18	38 Gy/19f	50.4 Gy/28f	2574	3
19	38 Gy/19f	50.4 Gy/28f	2574	3

*F* fractions, *RT* radiation therapy, *RID* radiation-induced dermatitis.

**Figure 2:**
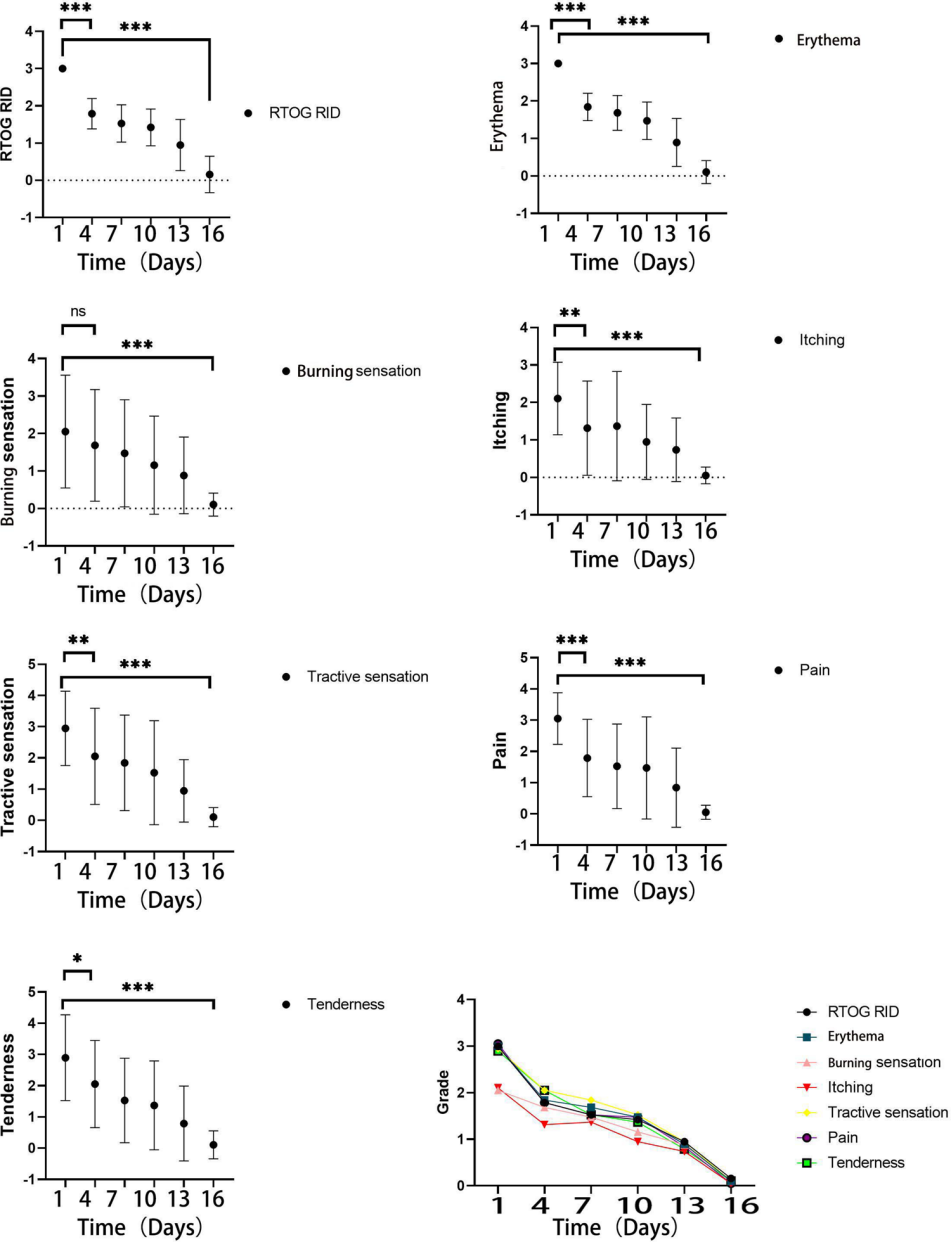
Effect of EGCG treatment for RID and related symptoms.

As the EGCG treatment was performed continuously, all these grade III RID reactions were significantly decreased to grade I or grade II RID at 1 weeks after use of EGCG (*p* < 0.001). The RTOG RIDs were recorded every day from the first usage of EGCG. After 15 days follow up, one patient reported grade II RID and one patient reported grade I RID. Other patients all reported no RID after EGCG treatment (*p* < 0.001). The decreasing trend of RID was shown in Fig. 2.

**Figure 3:**
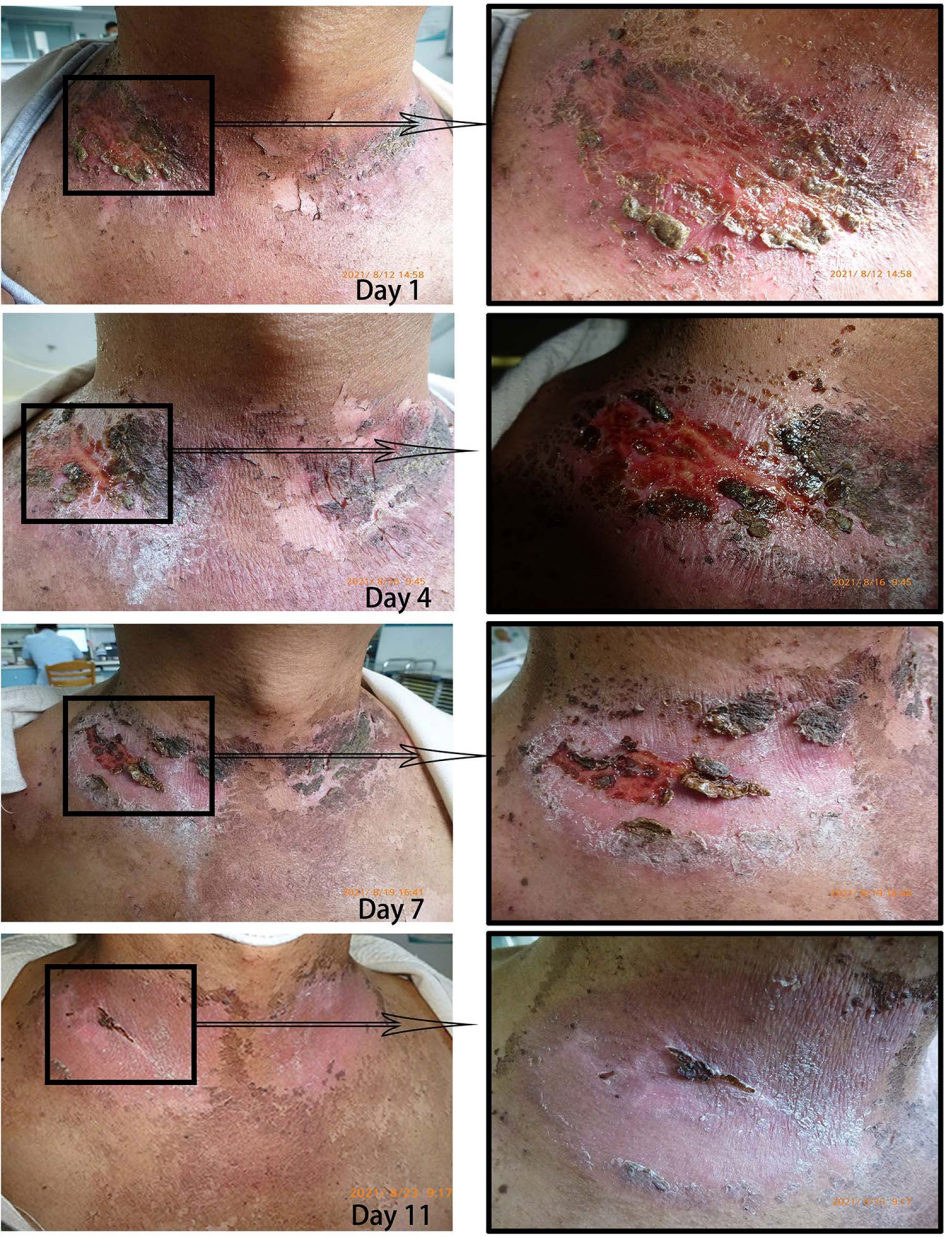
RID reaction of a patient treated with EGCG. Related files.

Our research team also recorded other related symptoms. Significant relief can be observed in burning sensation (*p* < 0.001), tractive sensation (*p* < 0.001), tenderness (*p* < 0.001), erythema (*p* < 0.001), itching (*p* < 0.001) and pain (*p* < 0.001) at the last follow up of EGCG treatment. Details were shown in Fig. 2. The regression of patient-reported symptoms related to acute skin reactions did not seem to correlate with the onset time and the dose of EGCG. One representative case is shown in Fig. 3. No radiation therapy delay or interruption for all 19 patients.

### Safety of EGCG for treatment of grade III RID

The dose of EGCG solution began from 660 to 2574 μmol/L, no adverse events were observed and reported associated with EGCG. And also, no EGCG dose reduction because of adverse events of skin. Even for the skin ulcer after RT, EGCG was well tolerated for all patients. Thus, the highest dose of this Phase I trial (2574 μmol/L) was recommended for continuous Phase II trial for further evaluation.

## Discussion

Acute RID usually arise within 90 days of the initiation of therapy with the characteristics of skin dryness, hair loss, hyperpigmentation Dry desquamation, skin scaling and flaking moist desquamation^14,15^. When the cumulative radiation dose reaches 20–30 Gy, dry desquamation can be observed in patients^14,15^.

After the cumulative dose for the skin reach to 40 Gy or higher, a more severe RID can be observed in RT filed of skin. Such as edema, fibrinous exudates, and the potential for bullae formation. The damp and tender skin of RT field is painful, which is prone to infection. Radiotherapy may be interrupted due to grade II to III RID^14^.

No evidence-based standard care has been established for treat RID^16^. Our research team conducted phase I clinical trial for preventing acute radiation skin adverse events^14,15^. In this research, EGCG was proved to be safe for patients when the dose of EGCG escalation stopped at 660 mmol/L and no other reported acute toxicity was associated with EGCG. Patient reported RID related symptoms such as pain, burning, itching and tenderness were significantly decreased at 2 weeks after the end of radiotherapy^14^. Thus, our research concluded that EGCG might be effective in treating acute radiation adverse events. Based on previous researches, our research team conducted a double-blind, placebo-controlled, phase 2 randomized clinical trial to evaluate the efficacy of EGCG in preventing RID in patients with breast cancer receiving postoperative radiotherapy^12^. The results showed that prophylactic use of EGCG solution significantly reduced the incidence and severity of RID in patients receiving adjuvant radiotherapy for breast cancer.

However, previous studies only enrolled grade I to II RID, and did not included grade III or higher RID of patients during RT^12,14,15^. There is still no standard treatment for patients with ulcer and skin damage exudation during and after the RT period. Therefore, with the data support of our previous research, our research team further explored whether EGCG is safe and can have expected benefits in treating RID of more than grade III.

Patients received RT field including supraclavicular area or chest wall were observed and enrolled in our research when grade III was observed in those patients. No adverse events associated with EGCG was observed in those patients, even for the ulcer filed of skin. Results showed that symptoms of grade III RID were all reduced and improved significantly after usage of EGCG.

In preclinical trial conducted by professor Zhu found that pretreatment with EGCG significantly enhanced the viability of the human skin cells irradiated by X-rays, and decreased apoptosis induced by X-ray irradiation^17^. EGCG induced the expression of the cytoprotective molecule hemeoxygenase-1 in a dose-dependent manner via transcriptional activation. That might also be one of the mechanisms of EGCG in treating grade III RID. In our study, results showed that EGCG dose escalating to 2574 mm/L was a safe and effective dose in the treatment of grade III RID.

However, our study still had some limitations. The number of enrolled patients was small. What’s more, the safety and effectiveness need of EGCG still need further evaluation in prospective research.
